# Planar cell polarity gene expression correlates with tumor cell viability and prognostic outcome in neuroblastoma

**DOI:** 10.1186/s12885-016-2293-2

**Published:** 2016-03-31

**Authors:** Cecilia Dyberg, Panagiotis Papachristou, Bjørn Helge Haug, Hugo Lagercrantz, Per Kogner, Thomas Ringstedt, Malin Wickström, John Inge Johnsen

**Affiliations:** Childhood Cancer Research Unit, Department of Women’s and Children’s Health, Karolinska Institutet, Astrid Lindgren Children’s Hospital Q6:05, SE-171 76 Stockholm, Sweden; Neonatal Research Unit, Department of Women’s and Children’s Health, Astrid Lindgren Children’s hospital, Q2:07, Karolinska Institutet, SE-171 76 Stockholm, Sweden; Academic Primary Health Care Center, TioHundra AB, Box 905, SE-761 29 Norrtälje, Sweden; Department of Pediatrics, University-Hospital of Northern-Norway (UNN), 9037 Tromsø, Norway

**Keywords:** Wnt/PCP pathway, Neuroblastoma, Prickle1, Vangl2

## Abstract

**Background:**

The non-canonical Wnt/Planar cell polarity (PCP) signaling pathway is a major player in cell migration during embryonal development and has recently been implicated in tumorigenesis.

**Methods:**

Transfections with cDNA plasmids or siRNA were used to increase and suppress *Prickle1* and *Vangl2* expression in neuroblastoma cells and in non-tumorigenic cells. Cell viability was measured by trypan blue exclusion and protein expression was determined with western blotting. Transcriptional activity was studied with luciferase reporter assay and mRNA expression with real-time RT-PCR. Immunofluorescence stainings were used to study the effects of *Vangl2* overexpression in non-tumorigenic embryonic cells. Statistical significance was tested with *t*-test or one-way ANOVA.

**Results:**

Here we show that high expression of the PCP core genes *Prickle1* and *Vangl2* is associated with low-risk neuroblastoma, suppression of neuroblastoma cell growth and decreased Wnt/β-catenin signaling. Inhibition of Rho-associated kinases (ROCKs) that are important in mediating non-canonical Wnt signaling resulted in increased expression of *Prickle1* and inhibition of β-catenin activity in neuroblastoma cells. In contrast, overexpression of *Vangl2* in MYC immortalized neural stem cells induced accumulation of active β-catenin and decreased the neural differentiation marker Tuj1. Similarly, genetically modified mice with forced overexpression of *Vangl2* in nestin-positive cells showed decreased Tuj1 differentiation marker during embryonal development.

**Conclusions:**

Our experimental data demonstrate that high expression of *Prickle1* and *Vangl2* reduce the growth of neuroblastoma cells and indicate different roles of PCP proteins in tumorigenic cells compared to normal cells. These results suggest that the activity of the non-canonical Wnt/PCP signaling pathway is important for neuroblastoma development and that manipulation of the Wnt/PCP pathway provides a possible therapy for neuroblastoma.

**Electronic supplementary material:**

The online version of this article (doi:10.1186/s12885-016-2293-2) contains supplementary material, which is available to authorized users.

## Background

Neuroblastoma, an embryonic tumor of the peripheral sympathetic nervous system is the most common and deadly tumor of childhood [1]. These tumors are clinically and biologically heterogeneous ranging from highly proliferative tumors that undergo spontaneous apoptosis with little or no treatment to highly malignant metastasizing tumors that are difficult to cure with current treatment strategies [1, 2]. Primary neuroblastoma occurs in the adrenal medulla and the paraspinal sympathetic ganglia and likely derives from cells within the neural crest [3]. The neural crest is a transient population of multipotent migratory cells emerging from the dorsal neural tube and gives rise to a wide variety of different cells including those of the sympathetic lineage [4]. During formation of the neural crest a combined action of fibroblast growth factor, bone morphogenetic protein and Wingless (Wnt) signaling is required to specify the location of neural crest cells at the neural plate border [5]. Neural crest cells migrate from the neural plate in a process equivalent to epithelial-mesenchymal transition (EMT) in which the cells locomotion, orientation and polarization are controlled mainly by the non-canonical Wnt/Planar cell polarity (PCP) signaling cascade [6]. Inappropriate neural crest cell migration and differentiation may lead to ectopic tissue formation and is associated with a number of diseases including neuroblastoma [7].

The most distinct marker for poor survival in neuroblastoma is *MYCN* gene amplification which is found in approximately 40 % of high-risk neuroblastomas [8]. However, high-risk neuroblastomas without *MYCN* gene amplification frequently display increased levels of active β-catenin and activation of canonical Wnt/β-catenin signaling [9]. The non-canonical Wnt/PCP core proteins Prickle1 and Van Gogh-like 2 (Vangl2) have recently been shown to attenuate with canonical Wnt/β-catenin signaling partly by destabilization of β-catenin [10, 11]. This has led to the suggestion that these proteins may behave as tumor suppressors in certain cancers [10].

In this study we have investigated the effects of manipulating the expression levels of PCP proteins in neuroblastoma cells. We analyzed neuroblastoma expression cohorts and show that high expression of the PCP proteins *Prickle1* and *Vangl2* correlates with low-risk disease and patient survival. Genetic knock-down of the core PCP genes *Prickle1* or *Vangl2* resulted in increased growth of neuroblastoma cells and increased active β-catenin levels, while overexpression had the opposite effect. Also pharmacological inhibition of Rho-associated coiled-coil kinase (ROCK), an important downstream effector of non-canonical Wnt signaling resulted in increased expression of *Prickle1* and reduced levels of active β-catenin. In contrast, in non-tumorigenic neural stem cells *Vangl2* knockdown decreased cell growth and increased differentiation while overexpression showed impaired differentiation. These results were also confirmed in transgenic mouse embryos that are genetically modified to overexpress Vangl2 in nestin-positive cells.

## Methods

### Cell lines

Neuroblastoma cells were cultured in RPMI 1640 (SK-N-AS, SK-N-BE (2), SK-N-DZ, SK-N-FI, IMR-32, Kelly, SH-EP1 and SK-N-SH) or Dulbecco’s modified Eagle’s medium (DMEM)/F12 (SH-SY5Y), supplemented with 10 % fetal bovine serum (FBS), 2 mM L-glutamine, and antibiotics (streptomycin and penicillin) from GIBCO (Life Technologies, Thermo Fisher Scientific Inc., Waltham, MA USA) [12]. The MYC immortalized neural stem cells line C17.2 [13, 14] was cultivated in DMEM supplemented with 10 % FBS, 5 % horse serum, 2 mM L-glutamine and antibiotics (GIBCO). Experiments were performed in Opti-MEM (GIBCO) supplemented with glutamine and antibiotics, except for transfection experiments, which were performed without antibiotics. The identities of the neuroblastoma cell lines were verified by short tandem repeat genetic profiling using the AmpFlSTR Identifiler PCR Amplification Kit (Applied Biosystems, Life Technologies, Thermo Fisher Scientific Inc., Stockholm, Sweden) in October 2015 and all cell lines were used in passages below 25.

### Transfections

Cells were transfected using Lipofectamine 2000 (Invitrogen, Life Technologies) according to the manufacturer’s instructions and incubated for 48 h before analysis. Expression plasmids for hPrickle1, hVangl2 and cDNA control were a kind gift (provided respectively by Dr. A. Bassuk at the University of Iowa and Dr. L. Braiterman at the Johns Hopkins University School of Medicine). Silencing RNA (siRNA) hairpins (Stealth siRNA duplex oligoribonucleotides) complementary to human *Prickle1* and *Vangl2* mRNAs were designed by Invitrogen. Alternative siRNA sequences (Santa Cruz Biotechnology, Dallas, Texas USA), complementary to human *Prickle1* and *Vangl2* mRNAs were used in confirmative transfection experiments. The siRNAs used were a pooled cocktail with three different siRNA sequences. β-catenin knockdown was achieved using the SignalSilence β-catenin kit (Cell Signaling Technology, Beverly, MA). Non-silencing siRNA was used as control (Cell Signaling Technology). The final concentration of RNA when added to the cells was 33 nM.

### Viability assay

The viability effects of PCP gene expression (siRNA/overexpression by cDNA) on neuroblastoma cells were determined using trypan blue exclusion and manually counting in microscope chambers. Briefly, cells were seeded in 25 cm^2^ culture flasks, allowed to attach overnight, and transfected with cDNA or siRNA constructs of the PCP gene of interest for 48 h. Cells were then harvested and counted. All viability experiments were repeated at least three times.

### Drug treatments

To inhibit ROCK cells were drug treated with HA1077 (Fasudil, LC laboratories, Boston USA) (dissolved in PBS, tested in 25 μM and 50 μM, 72 h) or Y27632 (LC laboratories) (dissolved in dimethyl sulfoxide, tested in 80 μM, 72 h) and then further analyzed.

### Western blotting

Harvested cell pellets were lysed for 15 min with ice-cold lysis buffer (50 mM Tris–HCl pH 7.4, 150 mM sodium chloride, 0.1 % SDS, 1 mM EDTA, 1× Roche protease inhibitor cocktail). For Western blot analyses, samples containing equal amounts of protein were separated by gel electrophoresis and electroblotted onto Hybond-P membranes (Amersham Pharmacia, Cleveland, OH USA). Blots were blocked with 5 % skim milk, followed by incubation with antibodies specific for anti-Vangl2 (1:1000, R&D Systems, Minneapolis, MN USA), anti-Prickle1 (1:1000; Santa Cruz Biotechnology), anti-full length β**-**catenin (1:1000, Cell Signaling Technology)**,** anti-active β-catenin (1:1000, clone 8E7, Millipore, Solna, Sweden), anti-Axin2 (1:1000, Cell Signaling Technology), anti-β-actin (1:5000, Cell Signaling Technology) and anti-GADPH (1:10000, Millipore). Blots were further incubated with goat anti-rabbit or anti-mouse secondary antibody conjugated to horseradish peroxidase (Amersham) accordingly to manufactures instruction and developed on Kodak hyperfilm. Quantification of blots were done with densitometry measurements in ImageJ [15].

### Real-time RT-PCR analyses

The mRNA expression levels of *Prickle1*, *Vangl2* and endogenous housekeeping genes were quantified using TaqMan® technology on an ABI PRISM 7500 sequence detection systems (Applied Biosystems) or performed with Power SYBR Green master mix (Life technologies) on a 7300 Real-Time PCR system (Life technologies). The TaqMan® sequence-specific primers included Vangl2 (Hs00393412_m1), Prickle1 (Hs01055551_m1), and 18S ribosomal RNA (Hs99999901_s1) (Applied Biosystems). Primer sequences for SYBR Green were as followed: Vangl2: F: TCTACAACGTTGGCCATCTCAGC and R: ACACCTTGAAGCCAGACACTTTC. Prickle1: F: TGCTCAGCGGAAGAAAGAAGCAC and R: AGCATGCATGACTGCTCTGGAC. GAPDH: F: GAAATCCCATCACCATCTTCCAGG and R: GAGCCCCAGCCTTCTCCATG.

To create a standard curve for relative quantification we used cDNA synthesized from 1 μg RNA. Total RNA was prepared from cultured cells using the RNeasy Mini Kit (Qiagen AB, Sollentuna, Sweden) or TRIzol reagent (Life technologies) according to manufacturers protocol. The cDNA synthesis was performed using High capacity RNA-to-cDNA kit (Applied Biosystems) or High capacity cDNA reverse transcription kit (Life technologies). All real-time RT–PCR experiments included a no template control and were performed in triplicate.

### Luciferase reporter assay

Cells were seeded in 24-well plates, left to attach and transfected with a T-cell factor/lymphoid enhancing factor (TCF/LEF) reporter plasmid (Super 8× TOPFlash; 400 ng), a Renilla-Luc plasmid (40 ng) and siRNA constructs for *Prickle1* or *Vangl2* using Lipofectamine 2000 (Invitrogen). Alternatively cells were transfected with the TCF/LEF reporter plasmid and the Renilla-Luc plasmid and 24 h later, drug treated with the ROCK inhibitor HA1077. A Dual Luciferase Assay Kit (Promega, Fitchburg, Wisconsin USA) and a luminometer (Perkin Elmer, Waltham Massachusetts USA) were used to measure luminescence. The values were normalized to the Renilla reporter before calculating relative levels.

### Generation of the Vangl2-HA and nestin-Vangl2 transgenic embryos

A 1566-bp fragment spanning the open reading frame of Vangl2 and flanked by *Xho*I and *Hind*III sites was generated by PCR from a cDNA clone containing the Vangl2 coding sequence [I.M.A.G.E. Consortium (LLNL) cDNA CloneID 6509008 [16]] purchased from RZPD (www.rzpd.de; RZPD CloneID IMAGp998J1714075Q3). It was then inserted into the *Xho*I and/or *Hind*III site of the pcDNA3-HA expression vector or the *Not*I site of the human nestin (hnestin) 1852 vector [17, 18]. The expression cassette, hnestin 1852/tk promoter Vangl2 ORF was used for pronuclear injection of fertilized mouse oocytes. The transgenic mouse embryos were generated at the Karolinska Center for Transgene Technologies using standard techniques. Shortly, oocytes from female B6D2F1 (F1 strain of C57B1/6 × DBA2) mated with male B6D2F1, were retrieved from the oviducts and the DNA construct was injected into the male pronucleus. Fertilized zygots were then reimplanted into a pseudopregnant foster female (NMRI strain). Pregnant females with embryos of E8.5 or E9.5 were sacrificed by spinal dislocation, and the embryos were rapidly dissected out. Yolk sac DNA was used to genotype transgenic mouse embryos. To identify transgenics, PCR was performed with a sense primer complementary to human nestin intron 2 combined with an antisense primer complementary to the Vangl2 ORF. Mice were kept at maximum of six per cage and were given water and food *ad libitium*. The animal experiment was recorded according to the guidelines given in the ARRIVE protocol [19]. All animal experiments were approved by the Northern Stockholm ethics committee for animal research (N163/03 and N142/06), appointed and under the control of the Swedish Board of Agriculture and the Swedish Court. The animal experiments presented herein were in accordance with national regulations (SFS 1988:534, SFS 1988:539 and SFS 1988:541) and European Communities Council guidelines (directive 86/609/EEC).

### Immunohistochemistry

Embryos were fixed overnight in 4 % paraformaldehyde in PBS (pH 7.4) and cryoprotected overnight in 30 % sucrose in phosphate-buffered saline (PBS). The embryos were then embedded in mounting medium (Tissue-Tek) and rapidly frozen. 12-μm sections were collected in a cryostat (Leica CM3050S; Leica Microsystems Nussloch GmbH, Germany) and blocked in 5 % goat serum (Jackson Immunoresearch Laboratories, West Grove, PA), and 0.03 % Triton X-100 (Amersham) in PBS for 45 min followed by overnight incubation with primary antibodies in PBS with 5 % goat serum and 0.03 % Triton X-100. The following antibodies and dilutions were used: mouse anti-beta- III/Tuj1 (1:500, Covance, Princeton, NJ, United States of America), rabbit anti-HA (1:200, Sigma), rabbit anti-phospho-Histone-3 (1:2000, Merck Chemicals, Merck Chemicals and Life Science AB, Stockholm, Sweden). Followed by incubation 1 h room temperature with the appropriate Alexa fluor-conjugated secondary antibodies (Molecular Probes, Invitrogen) at a 1:400 dilution in PBS with 5 % goat serum and 0.03 % Triton X-100. Finally sections were rinsed and mounted in Vectashield Hard Set mounting medium.

C17.2 cells were fixed with 4 % paraformaldehyde, permeabilized and blocked in 7 % non-fat dry milk and 0.1 % Triton X-100 in PBS. Primary antibodies were incubated at 4 °C overnight. Primary antibodies used were anti-β-catenin (1:200, Merck Chemicals) and mouse anti-beta- III/Tuj1 (1:500, Covance, Princeton, NJ, United States of America). Followed by incubation 1 h room temperature with the appropriate Alexa fluor-conjugated secondary antibodies diluted in PBS and mounted in Vectashield Hard Set mounting medium. Fluorescent images were captured with a Nikon axiocam fluorescence microscope, 20× objective. Contrast images were acquired in a Nikon Eclipse TS100 microscope, 20× objective.

### Statistical analysis

Differences between two groups were determined using two-sided *t*-test and for three or more groups one-way ANOVA with Bonferroni post-test was used. Kaplan-Meier survival estimates and gene correlation graphs were extracted from the R2 database (R2: microarray analysis and visualization platform (http://r2.amc.nl)).

## Results

### Differential expression and interaction of PCP proteins in neuroblastoma

**Fig. 1:**
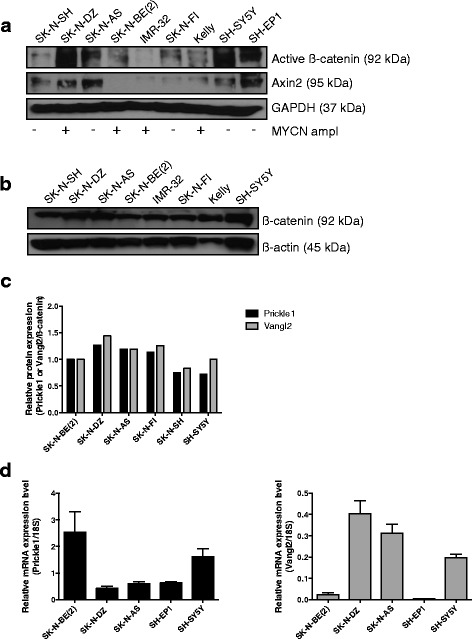
Active β-catenin, Prickle1 and Vangl2 are differently expressed in neuroblastoma cell lines. **a** Protein expression of active, de-phoshorylated β-catenin, the canonical Wnt/β-catenin target gene Axin2 and **b**. total β-catenin in neuroblastoma cell lines. SK-N-BE (2), SK-N-DZ, IMR-32 and Kelly are *MYCN* amplified neuroblastoma cells with high MycN expression. SH-SY5Y, SK-N-SH and SK-N-FI are non-*MYCN* amplified neuroblastoma cells expressing relatively low levels of MycN whereas, SK-N-AS and SHEP-1 do not show any MycN expression [33] **c**. Quantified protein expression (adjusted to β-actin) of Prickle1 and Vangl2 in neuroblastoma cell lines. Proteins were determined with western blotting. **d** mRNA expression in neuroblastoma cell lines SK-N-BE (2), SK-N-DZ, SK-N-AS, SH-EP1 and SH-SY5Y, assessed by quantitative real-time PCR, the data displayed is the mean ± S.D. of three determinations

The expression of active β-catenin was evaluated to screen for canonical Wnt signaling activity in neuroblastoma cell lines. All nine investigated neuroblastoma cell lines displayed active β-catenin, i.e. the dephosphorylated nuclear form, as well as the canonical Wnt target gene Axin2, regardless of MYCN gene amplification status [12] (Fig. 1a). However, the highest levels of active β-catenin were detected in SK-N-AS and SH-SY5Y cells that are either MYCN deficient or express low levels of MYCN, respectively. The *MYCN* amplified neuroblastoma cell lines IMR-32 and SK-N-BE (2) showed the lowest levels of active β-catenin (Fig. 1a). All neuroblastoma cell lines expressed abundant levels of total β-catenin (Fig. 1b)

Next, we investigated the level of the PCP core proteins Prickle1 and Vangl2 in neuroblastoma cells. Protein expression of Prickle1 and Vangl2 were detected in all tested neuroblastoma cell lines (Fig. 1c). We normalized the native expression levels against β-actin and compared the expression levels of the PCP proteins Prickle1 and Vangl2 against active β-catenin. Real-time quantitative PCR demonstrated that *Prickle1* expression was inversely correlated to active β-catenin/Axin2 levels in neuroblastoma cells (Fig. 1c, d). Neither Vangl2 protein nor mRNA displayed any correlation to active β-catenin (Fig. 1c, d).

### Expression of PCP core genes correlates with neuroblastoma survival

**Fig. 2:**
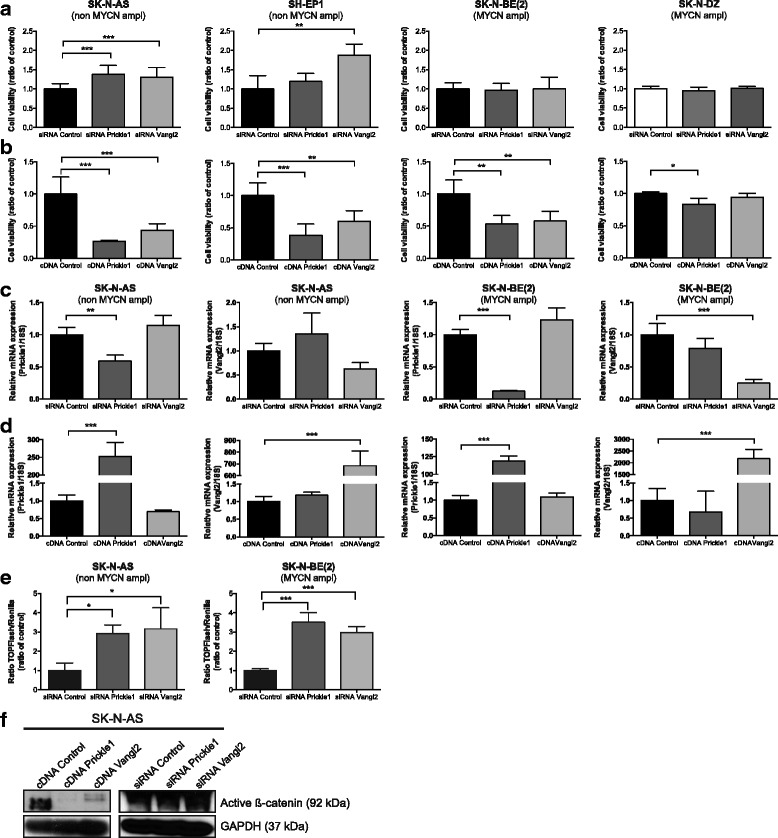
Knockdown and overexpression of *Prickle1* and *Vangl2* alter neuroblastoma cell viability and affect β-catenin expression. **a** Transfection with siRNA against *Prickle1* and *Vangl2* resulted in a significant increase of cell viability compared to control cells transfected with scrambled siRNA sequence (48 h) in SK-N-AS and SH-EP1, while no effects were observed in SK-N-BE (2) and SK-N-DZ cells (one-way ANOVA with Bonferroni post-test, SK-N-AS: *P <* 0.0001 control vs Prickle1 *P <* 0.0001, control vs Vangl2 *P =* 0.0003; SH-EP1: *P =* 0.0023 control vs Vangl2 *P =* 0.0016). **b** Overexpression of *Prickle1* and *Vangl2* in SK-N-AS, SH-EP1, SK-N-BE (2) and SK-N-DZ decreased cell viability significantly, compared control transfected cells (one-way ANOVA with Bonferroni post-test, SK-N-AS: *P <* 0.0001 control vs Prickle1 *P <* 0.0001, control vs Vangl2 *P =* 0.0003, SH-EP1: *P =* 0.0004 control vs Prickle1 *P =* 0.0002, control vs Vangl2 *P =* 0.013, SK-N-BE (2): *P =* 0.0014 control vs Prickle1 *P =* 0.019 control vs Vangl2 *P =* 0.0025 and SK-N-DZ: *P =* 0.020, control vs Prickle1 *P =* 0.014). Cell viability was assessed by manually courting in microscope chamber. Mean with SD are displayed, the experiments were repeated with similar results. **c**, **d** mRNA expression of *Prickle1* and *Vangl2* after knockdown and overexpression of *Prickle1* or *Vangl2* in neuroblastoma cells. All transfections induced significant up-/downregulation of its target gene except from siRNA *Vangl2* in SK-N-AS (one-way ANOVA with Bonferroni post-test: SK-N-AS: control vs siRNA Prickle1 *P =* 0.0024, control vs cDNA Prickle1 *P <* 0.0001 and control vs cDNA Vangl2 *P <* 0.0001, SK-N-BE (2): control vs siRNA Prickle1 *P <* 0.0001, control vs siRNA Vangl2 *P =* 0.0002, control vs cDNA Prickle1 *P <* 0.0001 and control vs cDNA Vangl2 *P =* 0.0003). Data displayed is the mean ± S.D. of three determinations, assessed by quantitative real-time PCR. **e** The transcriptional activity of β-catenin measured as TOPflash luciferase activity was significantly induced after *Prickle1* or *Vangl2* knockdown (one-way ANOVA with Bonferroni post-test, SK-N-AS *P =* 0.0194, control vs siRNA Prickle1 *P =* 0.034, control vs siRNA Vangl2 *P =* 0.021 and SK-N-BE (2) *P =* 0.0003, control vs siRNA Prickle1 *P =* 0.0002, control vs siRNA Vangl2 *P =* 0.0009). Values are mean ± S.D., the experiment was repeated twice. **f** Protein expression of active β-catenin after upregulated or downregulated Prickle1 or Vangl2 in SK-N-AS (48 h transfection), determined by western blotting. *P < 0.05, **P < 0.01, ***P < 0.001

To functionally analyze the impact of the expression level of PCP core genes in neuroblastoma, we transiently transfected SK-N-AS, SH-EP1, SK-N-BE (2) and SK-N-DZ neuroblastoma cells with siRNA or cDNA expression constructs for *Prickle1* or *Vangl2*. Knockdown of *Prickle1* or *Vangl2* by siRNA resulted in an increase of neuroblastoma cell growth in SK-N-AS cells (Prickle1, 138 % and Vangl2, 131 %) and SH-EP1 (Prickle1 119 % and Vangl2 188 %) compared to control cells treated with a scrambled siRNA sequence, while no changes were detected in the *MYCN* amplified neuroblastoma cell lines, SK-N-BE (2) and SK-N-DZ (Fig. 2a). To minimize the risk for eventually off-target effects caused by the pooled siRNA’s, we repeated the experiments using alternative siRNA’s targeting Prickle1 and Vangl2. Similar results on cell viability were obtained for knockdown of *Prickle1* or *Vangl2* in SK-N-AS and SK-N-BE (2) cells (Additional file 1: Figure S1). Overexpression of *Prickle1* or *Vangl2* significantly inhibited neuroblastoma cell growth compared to cDNA control transfected cells in SK-N-AS (Prickle1 26 % and Vangl2 44 %), SH-EP1 (Prickle1 38 % and Vangl2 60 %), SK-N-BE (2) (Prickle1 53 % and Vangl2 58 %) and SK-N-DZ (Prickle1 83 % and Vangl2 94 %) (Fig. 2b).

Knockdown and overexpression were confirmed with real-time quantitative PCR in SK-N-AS and SK-N-BE (2). All siRNA/cDNA transfection induced significant decrease or increase of its target gene except from siRNA against Vangl2 in SK-N-AS cells (Fig. 2c, d). The mRNA expression of *Prickle1* was not affected after knockdown or overexpression of *Vangl2* and similarly, the mRNA expression of *Vangl2* was not affected after knockdown or overexpression of *Prickle1* in SK-N-AS or SK-N-BE (2) cells (Fig. 2c, d).

### Altered expression of Prickle1 or Vangl2 affects active β-catenin activity in neuroblastoma cells

To investigate if Prickle1 and Vangl2 affected canonical Wnt signaling we studied the β-catenin transcriptional activity after repressed expression of *Prickle1* and *Vangl2* in SK-N-AS and SK-N-BE (2) cells. Knockdown of *Prickle1* or *Vangl2* induced a significant increase in TOPFlash luciferase reporter activity i.e. β-catenin transcriptional activity, compared to siRNA control transfected neuroblastoma cells (Fig. 2e). This increase in active β-catenin was confirmed with western blot in SK-N-AS cells (Fig. 2f). Furthermore, the protein expression of active β-catenin was correspondently decreased in cDNA transfected SK-N-AS cells compared to cDNA control cells (Fig. 2f).

### Alterations in the PCP signaling pathway have influence on the activity of active β-catenin in neuroblastoma cells

**Fig. 3:**
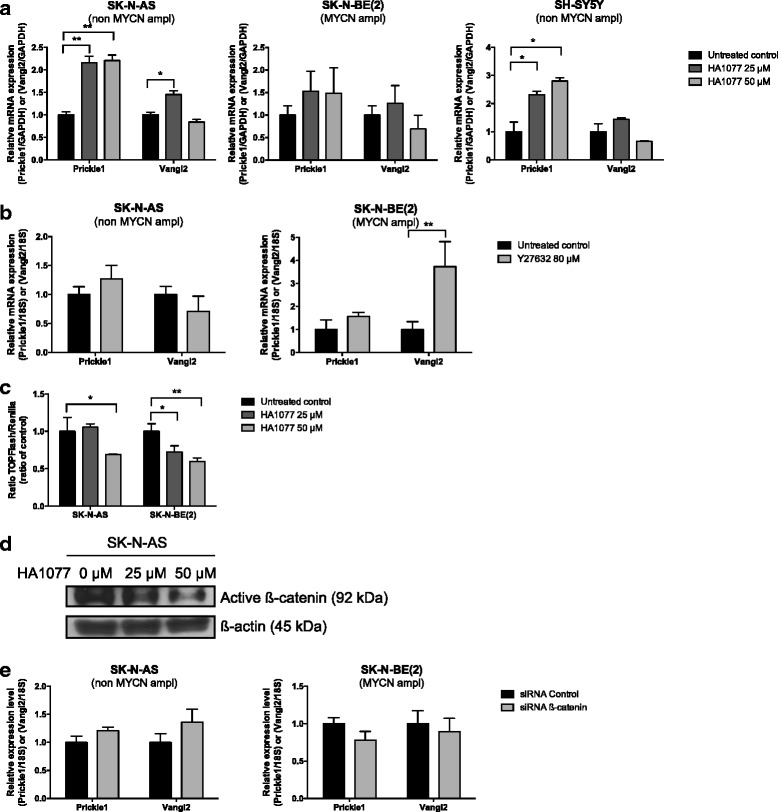
Inhibition of Wnt/PCP downstream effector ROCK increases *Prickle1* expression and represses active β-catenin. **a**, **b** mRNA expression of *Prickle1* and *Vangl2* after treatment with ROCK inhibitor HA1077 or Y27632 for 72 h; results showed a consistent increase in *Prickle1* expression (one-way ANOVA with Bonferroni post-test, SK-N-AS Prickle1: *P =* 0.0017, control vs HA1077 50 μM *P =* 0.0023, control vs HA1077 50 μM *P =* 0.0021, Vangl2: *P =* 0.0061, control vs HA1077 50 μM *P =* 0.017 and SH-SY5Y Prickle1 *P =* 0.019, control vs HA1077 50 μM *P =* 0.035, control vs HA1077 50 μM *P =* 0.020 and *t*-test, SK-N-BE (2) Vangl2 control vs Y27632 80 μM *P =* 0.0015). Expression of mRNA (relative to the vehicle treated control normalized to the mean expression of the housekeeping genes) was determined by real-time RT-PCR, means with S.D. of triplicates are displayed. **c** Transcriptional activity of β-catenin after HA1077 exposure; cells were transfected with a TCF/LEF luciferase reporter construct and treated with HA1077 (25 or 50 μM). TOPFlash-dependent activity was significantly reduced as compared with the control (one-way ANOVA with Bonferroni post-test: SK-N-AS *P =* 0.0144, control vs HA1077 50 μM *P =* 0.029 and SK-N-BE (2) *P =* 0.0023, control vs HA1077 50 μM *P =* 0.012, control vs HA1077 50 μM *P =* 0.0017). Data represent the mean and SD of three determinations and the experiment was repeated twice. **d** Protein expression of active β-catenin following HA1077 exposure (96 h, HA1077 25 or 50 μM), determined by western blotting. **e **mRNA expression of *Prickle1* and *Vangl2* after knockdown of *β-catenin *in neuroblastoma cells SK-N-AS and SK-N-BE(2), no significant changes were observed. *P < 0.05, **P < 0.01

To investigate if inhibition of downstream non-canonical Wnt/PCP signaling could influence Prickle1 and Vangl2 expression, we used the ROCK inhibitors HA1077 and Y27632. Treatment of SK-N-AS, SK-N-BE (2) or SH-SY5Y with HA1077 resulted in increased mRNA expression of *Prickle1*, but there was no consistent impact on mRNA expression of *Vangl2* (Fig. 3a). Similar results were obtained in SK-N-AS and SK-N-BE (2) using Y27632 (Fig. 3b). HA1077 50 μM significantly affected the cell viability in SK-N-AS (cell number 36 % of untreated control) and SH-SY5Y (44 % of untreated control). In SK-N-BE (2) both tested concentrations of HA1077 decreased the cell viability (25 μM: 65 % and 50 μM: 27 % of untreated control). Y27632 80 μM induced no effects on cell viability in any of the tested cell lines. Further, treatment with HA1077 showed a concentration dependent decrease in TOPFlash reporter activity i.e. β-catenin transcriptional activity, compared to untreated neuroblastoma cells (Fig. 3c). The inhibitory effect of HA1077 on β-catenin expression was further verified in SK-N-AS cells by western blotting (Fig. 3d). To investigate possible feedback effects between β-catenin and *Prickle1* and *Vangl2*, we studied the effects on *Prickle1* and *Vangl2* after β-catenin knockdown in SK-N-AS and SK-N-BE (2) cells. No significant effects were observed on the mRNA expression levels (Fig. 3e).

### Vangl2 alterations affect cell growth, differentiation and active β-catenin expression in neural stem cells in vitro

**Fig. 4:**
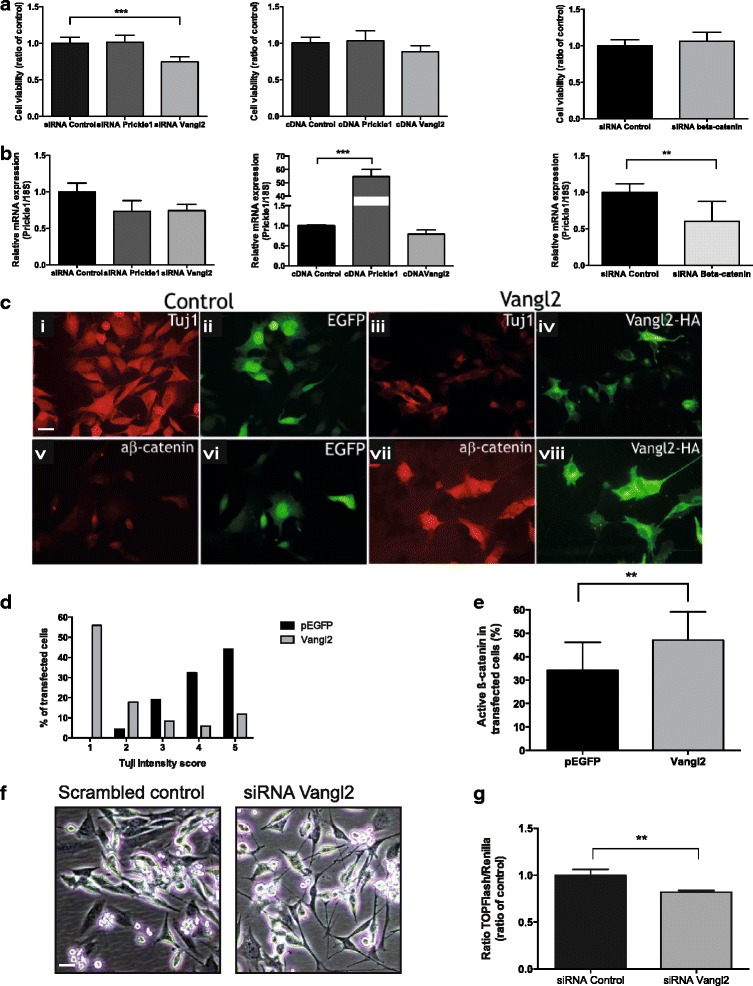
Vangl2 alterations affect cell growth, differentiation and active β-catenin expression in neural stem cells in vitro*.* **a** siRNA against *Vangl2* induced a significant decrease of cell viability compared to control cells transfected with a control siRNA sequence in C17.2 cells (one-way ANOVA with Bonferroni post-test, control vs siRNA Vangl2 *P =* 0.0004). siRNA against *Prickle1*, siRNA against *β-catenin*, cDNA for *Prickle1* or cDNA for *Vangl2* caused no change in cell viability. **b** The mRNA expression of *Prickle1* after siRNA or cDNA transfection of *Prickle1*, *Vangl2* or *β-catenin*. Only cDNA *Prickle1* and siRNA *β-catenin* induced significant changes in *Prickle1* mRNA expression (one-way ANOVA with Bonferroni post-test, control vs cDNA *Prickle1 P <* 0.0001, control vs siRNA β-catenin *P =* 0.0043). The mRNA expression of *Vangl2* was below the detection limit. Expression was determined with quantitative real-time PCR, mean with S.D. of three determinations are displayed. **c**-**e** *Vangl2* overexpression reduced the frequency of Tuj1 positive C17.2 cells (C, compare i and iii). For quantification 68 control and 84 Vangl2 cells were scored between 1 (no Tuj1 labeling) and 5 (very high labeling). All control transfected cells were Tuj1 positive (score 2–5). Contrary, more than half (56 %) of the Vangl2 transfected cells did not display any Tuj1 label (score 1) **d**. Vangl2 increased active β-catenin in the nucleus of C17.2 cells (C, compare v and vii). Active β-catenin was found in the nuclei of 33 % of the enhanced green fluorescent protein and 48 % of the HA-Vangl2 (*t*-test, *P =* 0.0037) **e**, mean with S.D. are shown. Scale bar: 10 μM. **f** *Vangl2* knockdown in C17.2 cells induced neural outgrowth, morphology consistent with increased differentiation compared to control transfected cells. Scale bar: 10 μM. **g** The transcriptional activity of β-catenin measured as TOPflash luciferase activity was significantly reduced after *Vangl2* knockdown in C17.2 cells (*t*-test, *P =* 0.0092). **P < 0.01, ***P < 0.001

To study the role of *Prickle1* and *Vangl2* in non-tumorigenic embryonic cells we used MYC immortalized neural stem cells, C17.2 [13, 14]. We performed transiently transfections with siRNA or cDNA expression constructs for *Prickle1* or *Vangl2* to study the impact of cell growth. Only knockdown of *Vangl2* resulted in a significant change in cell viability. In contrast to neuroblastoma cells, siRNA against Vangl2 decreased the cell number in C17.2 compared to siRNA control (75 %; Fig. 4a). The mRNA expression of *Prickle1* was significantly increased after overexpression of *Prickle1*, but no effect was recorded after siRNA *Prickle1* transfection (Fig. 4b). The mRNA expression of *Vangl2* could not be quantified in C17.2 as the levels were under the detection limit. To study possible feedback effects between β-catenin and Prickle1 and Vangl2 in non-tumorigenic cells, β-catenin knockdown in C17.2 cells was performed. No significant effects were observed on cell viability but the mRNA expression level of *Prickle1* was significantly reduced in β-catenin siRNA transfected cells compared to siRNA control transfected cells (Fig. 4a, b). Moreover, overexpression of *Vangl2* in C17.2 cells increased the amount of active β-catenin and reduced the levels of the differentiation marker Tuj1 (Fig. 4c, d, e). Correspondingly, *Vangl2* knockdown induced neural outgrowth consistent with differentiation and a significant decrease in β-catenin-dependent transcriptional activity (Fig. 4f, g).

### Vangl2 overexpression impairs differentiation in non-tumorigenic cells during embryonic development in vivo

**Fig. 5:**
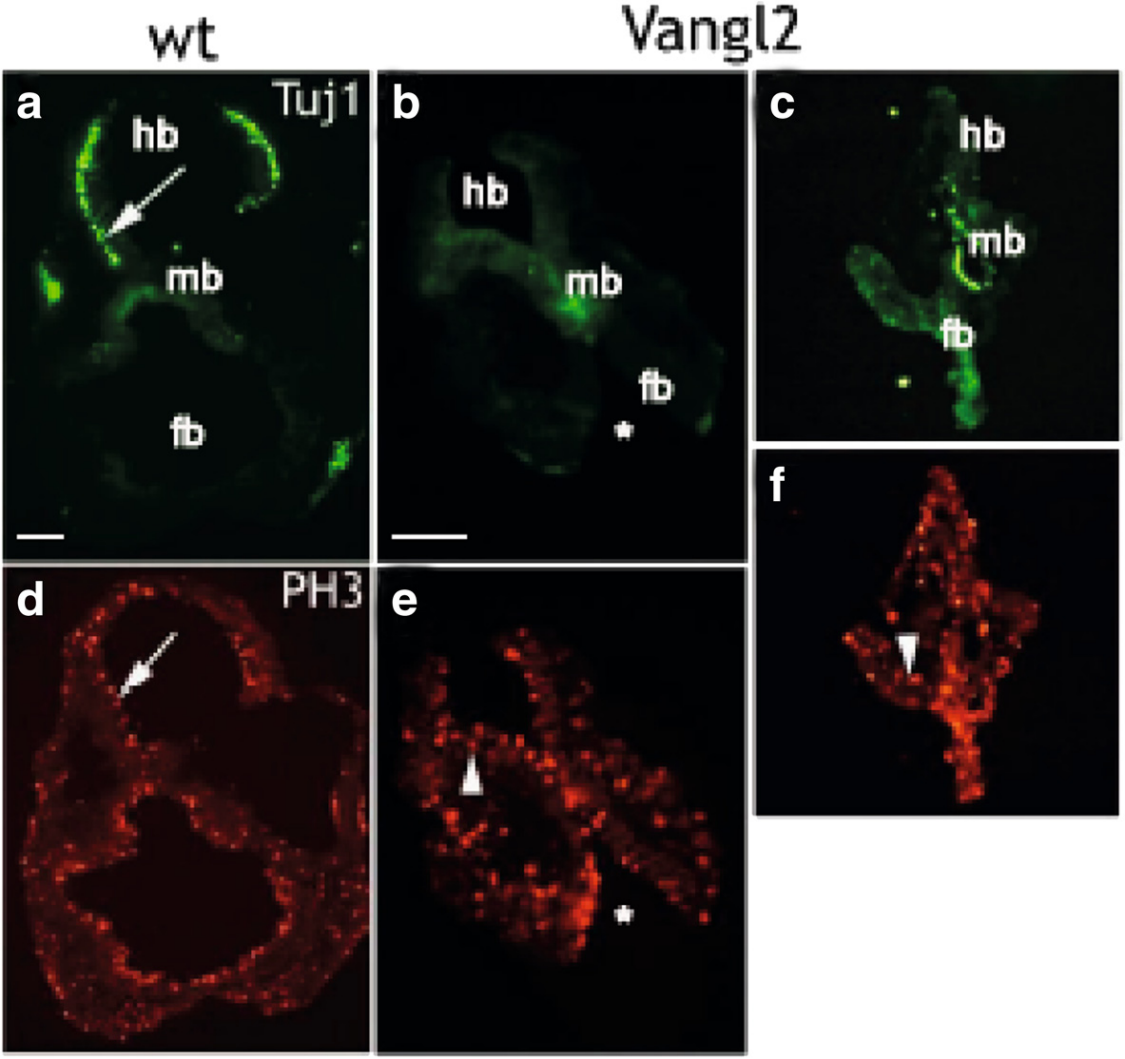
Vangl2 overexpression impairs differentiation during embryonic development. **a**-**f** Micrographs of 10 μm transversal tissue sections of neural mouse embryo E9.5 tissue labeled with antibodies against beta-tubulin-III (Tuj1, green) and phospho-Histone-3 (P-H3, red) and visualized with fluorescent conjugated secondary antibodies respectively. Wild-type E9.5 mouse embryos showed an even distribution of Tuj1^+^ neurons in the hindbrain part of the neuroepithelium (arrow in **a**), but were almost completely absent in the neuroepithelium of nestin-Vangl2 embryos (**b**) and (**c**). The labeling against M-phase marker PH3 was similar in the neuroepithelium of both wild type and transgenic sections (arrow in (**d**) and arrowhead in (**e**) and (**f**). Transgenic E9.5 Vangl2 embryos displayed impaired cranial neurulation (indicated with *[17];). Scale bars are 200 μm

To further investigate the role of Vangl2 in normal embryonic development we studied the effects of alteration in *Vangl2* in mice embryos in vivo. Transgenic overexpression of nestin-Vangl2 in mice embryos resulted in a drastic reduction of Tuj1+ neurons compared to wild type embryos E9.5. However, neuronal proliferation, assessed with cell cycle M-phase marker phosphorylated histone H3, was unaffected in transgenic mouse embryos compared to E9.5 wild type embryos (Fig. 5).

### High expression of Prickle1 and Vangl2 correlates with survival in neuroblastoma

**Fig. 6:**
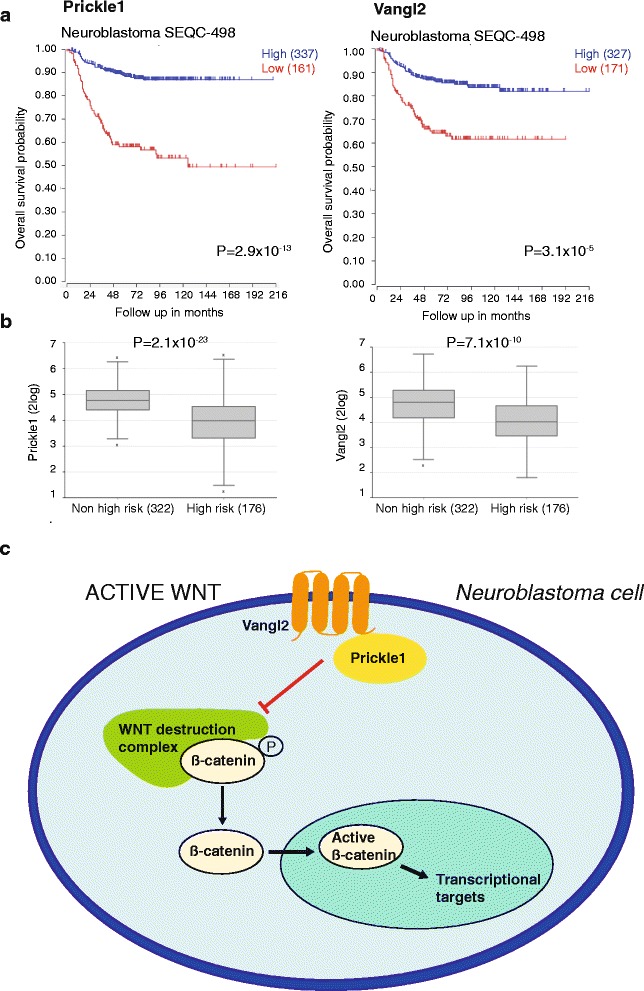
High expression of *Prickle1* and *Vangl2* correlates to survival and low-stage disease in neuroblastoma. **a** Kaplan-Meier survival estimates of high vs low expression of *Prickle1* and *Vangl2* in neuroblastoma expression cohorts analyzed using the microarray analysis and visualization platform (http://r2.amc.nl). **b** Box-plot of *Prickle1* and *Vangl2* expression correlated to neuroblastoma high-risk vs low/intermediate-risk disease stage. P-values were corrected for multiple testing (One-way Anova with Bonferroni post-test). **c** Schematic image over the possible interactions between Prickle1/Vangl2 and β-catenin in neuroblastoma

To investigate the clinical importance of PCP signaling in neuroblastoma we analyzed publicly available and validated cohorts of gene expression signatures. High expression of *Prickle1* and *Vangl2* corresponded to better survival and low-risk disease across different neuroblastoma expression array datasets (Fig. 6a, b, Additional file 1: Figure S2).

## Discussion

Neuroblastoma is a malignant neoplasm of the sympathetic nervous system that affects young children. Neuroblastoma originates from neural crest cells undergoing specification and differentiation. About 40 % of high-risk neuroblastoma contain gene amplification of the transcription factor *MYCN* often leading to high Mycn expression whereas about 6 % of neuroblastomas contain mutation of *ALK*, the majority seen in connection to *MYCN* gene amplification [2]. Also, other mutations like *PHOX2B* and *ATRX* as well as epigenetic aberrations have been reported in neuroblastoma [2]. However, although genetically unstable, the majority of neuroblastomas contain no such aberrations suggesting additional mechanisms for tumorigenesis. Also, the biological and clinical heterogeneity seen in neuroblastoma subtypes suggest the notion of neuroblastoma being a spectrum of diseases.

We show that core proteins within the non-canonical Wnt/PCP signaling cascade are differentially expressed in neuroblastoma and that the expression levels of these proteins affect neuroblastoma survival. Overexpression of *Prickle1* or *Vangl2* was coupled to decreased neuroblastoma growth and reduced expression of active β-catenin (summarized in Fig. 6c). This was also evident from analysis of expression arrays of primary neuroblastoma where high expression of *Prickle1* and *Vangl2* mRNA are significantly coupled to low-risk disease and good patient survival. Inhibiting the activity of ROCK1/2, important mediators of Rac/Rho signaling coupled to PCP activity also resulted in increased expression of *Prickle1* and inhibition of β-catenin activity. We used HA1077 and Y-27632 to inhibit the activity of ROCK in neuroblastoma cells. HA1077 was more effective in stimulating Prickle1 expression, inhibiting β-catenin activity and suppressing neuroblastoma growth as compared to Y-27632. This despite that both compounds binds to the ATP binding pocket of ROCKs with similar affinity (Ki 330 nM for HA1077 and 220–300 nM for Y-27632) leading to effective inhibition of the kinase activity [20]. Although off-target effects have been described for both compounds [21], we currently do not have any good explanation for these differences in the activity. In contrast to neuroblastoma cells, both non-tumorigenic neural cells and transgenic mouse embryos with overexpression of *Vangl2* in nestin-expressing cells showed increased active β-catenin and reduced differentiation. These results indicate that expression of PCP core proteins have different modes of action in neuroblastoma compared to non-tumorigenic neural cells and emphasize the complexity of the cell polarity network in different tissues. PCP signaling is fundamental for proper migration, polarity, locomotion and EMT of cells during embryonal development. Vangl2 is described to play an important role in developing CNS and mutations in Vangl2 are coupled to neural tube defects in mice and humans [22, 23]. Cell migration, polarity and EMT are processes also important during initiation, invasion and metastasis of tumor cells. Deregulated expression of molecules within the PCP signaling cascade has been shown in cancers of different origin including mammary gland tumors, hepatocellular carcinoma, colorectal, lung and prostate cancer and leukemia [10, 24–27]. However, contradictive reports indicate key molecules within the PCP signaling pathway as both tumor promoters and suppressors [10, 28]. For instance, *Vangl1* has been shown to both inhibit colorectal carcinoma metastasis in mice but also to promote and induce colorectal cancer metastasis in the same mouse model [29, 30]. Expression of the PCP core protein Prickle1 is downregulated in hepatocellular carcinoma whereas both Prickle1 and Vangl1 expression inhibit hepatocellular carcinoma growth in vitro [31, 32]. It is also demonstrated in hepatocellular carcinoma that high expression of Prickle1 inhibits active β-catenin though promoting ubiquitination and degradation of Dishevelled [31]. The observation in our study, that high expression of the PCP proteins *Prickle1* or *Vangl2* reduces the growth of neuroblastoma cells, further supports the notion that key proteins within the PCP signaling pathway may act as tumor suppressors. Similar to what we show for Prickle1, it has been reported that Scribble, another PCP protein, may inhibit β-catenin signaling and act as a tumor suppressor in mammary gland, prostate and lung cancer [10]. Taken together, accumulating evidence suggest that PCP proteins are important regulators of several cancer hallmarks and have potential both as diagnostic biomarkers for cancer aggressiveness and as future therapeutic targets.

## Conclusions

High expression of the PCP core proteins Prickle1 and Vangl2 reduce the growth of neuroblastoma cells and correspond to low-stage disease and patient survival. Our data also suggest that Prickle1 and Vangl2 expression have different modes of action in tumorigenic cells compared to normal cells. Our results suggest that the activity of the non-canonical Wnt/PCP signaling pathway is important for neuroblastoma development and that PCP proteins have potential both as diagnostic markers for neuroblastoma and as therapeutic targets.

## Additional file

Additional file 1: Knockdown of *Prickle1* or *Vangl2* alters neuroblastoma cell viability. Transfection of neuroblastoma cells using alternative siRNA’s against *Prickle1* and *Vangl2* resulted in a significant increase of cell viability compared to control cells transfected with scrambled siRNA sequence (48 h) in SK-N-AS. Also in SK-N-BE (2) an increase were recorded after siRNA against *Prickle1* (one-way ANOVA with Bonferroni post-test, SK-N-AS: *P <* 0.0001 control vs Prickle1 *P <* 0.0001, control vs Vangl2 *P <* 0.0001; SK-N-BE (2): *P =* 0.026: control vs Prickle1 *P =* 0.017). Figure S2: Correlation between expression of *Prickle1* and *Vangl2* and survival in neuroblastoma. **a**) Kaplan-Meier survival estimates of high vs low expression of *Prickle1* and *Vangl2* in neuroblastoma expression cohorts analyzed using the microarray analysis and visualization platform (http://r2.amc.nl). **b** Box-plot of *Prickle1* and *Vangl2* expression correlated to neuroblastoma disease stage (PPTX 486 kb)

## Abbreviations

DMEM: dulbecco’s modified eagle’s medium
E: embryonic day
EMT: epithelial-mesenchymal transition
fb: forebrain
FBS: fetal bovine serum
hb: hindbrain
mb: midbrain
PBS: phosphate-buffered saline
PCP: planar cell polarity
ROCK: rho-associated coiled-coil kinase
siRNA: silencing RNA
TCF/LEF: T-cell factor/lymphoid enhancing factor
Vangl2: van Gogh-like 2
Wnt: wingless
WT: wild-type
