# Supplementary material for: Planar cell polarity gene expression correlates with tumor cell viability and prognostic outcome in neuroblastoma
Source: BMC Cancer. 2016 Mar 31;16:259. doi: 10.1186/s12885-016-2293-2 (PMC4818482; doi:10.1186/s12885-016-2293-2)
Supplement: Additional file 1: — Knockdown of Prickle1 or Vangl2 alters neuroblastoma cell viability. Transfection of neuroblastoma cells using alternative siRNA’s against Prickle1 and Vangl2 resulted in a significant increase of cell viability compared to control cells transfected with scrambled siRNA sequence (48 h) in SK-N-AS. Also in SK-N-BE (2) an increase were recorded after siRNA against Prickle1 (one-way ANOVA with Bonferroni post-test, SK-N-AS: P < 0.0001 control vs Prickle1 P < 0.0001, control vs Vangl2 P < 0.0001; SK-N-BE (2): P = 0.026: control vs Prickle1 P = 0.017). Figure S2: Correlation between expression of Prickle1 and Vangl2 and survival in neuroblastoma. a) Kaplan-Meier survival estimates of high vs low expression of Prickle1 and Vangl2 in neuroblastoma expression cohorts analyzed using the microarray analysis and visualization platform (http://r2.amc.nl). b Box-plot of Prickle1 and Vangl2 expression correlated to neuroblastoma disease stage (PPTX 486 kb) [file 12885_2016_2293_MOESM1_ESM.pptx]

## Slide 1
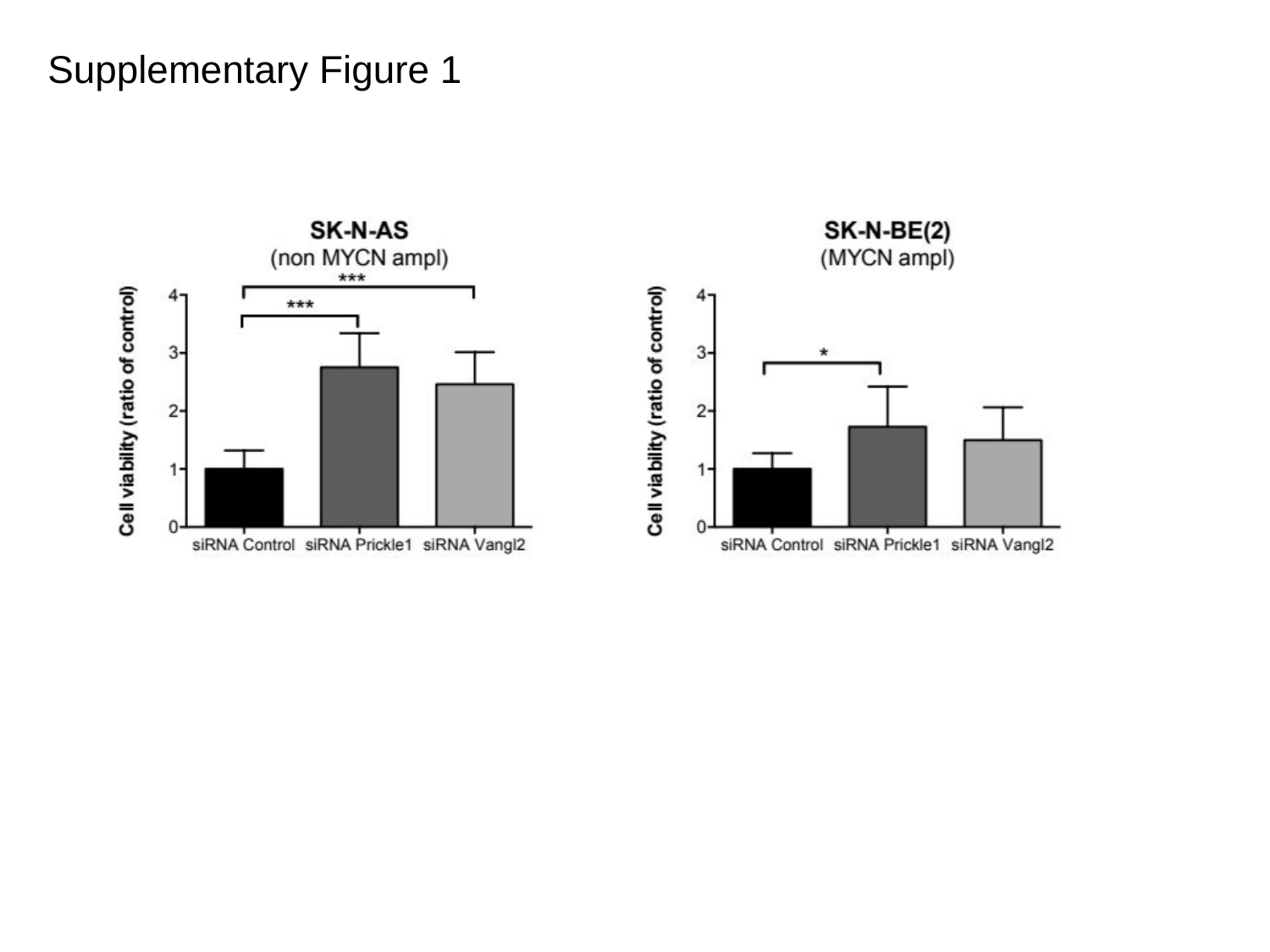

Supplementary Figure 1

## Slide 2
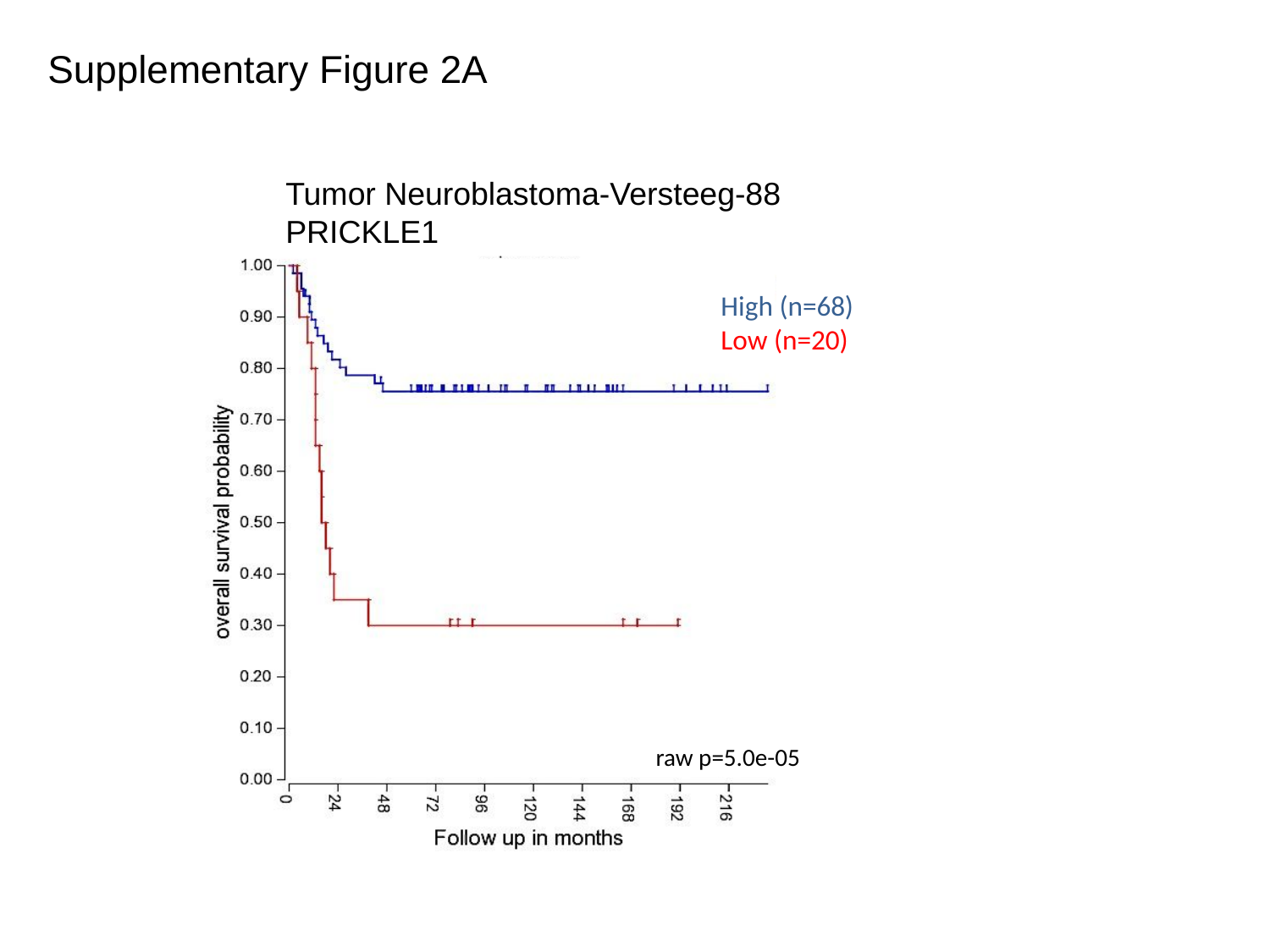

Supplementary Figure 2A
Tumor Neuroblastoma-Versteeg-88
PRICKLE1
High (n=68)
Low (n=20)
raw p=5.0e-05

## Slide 3
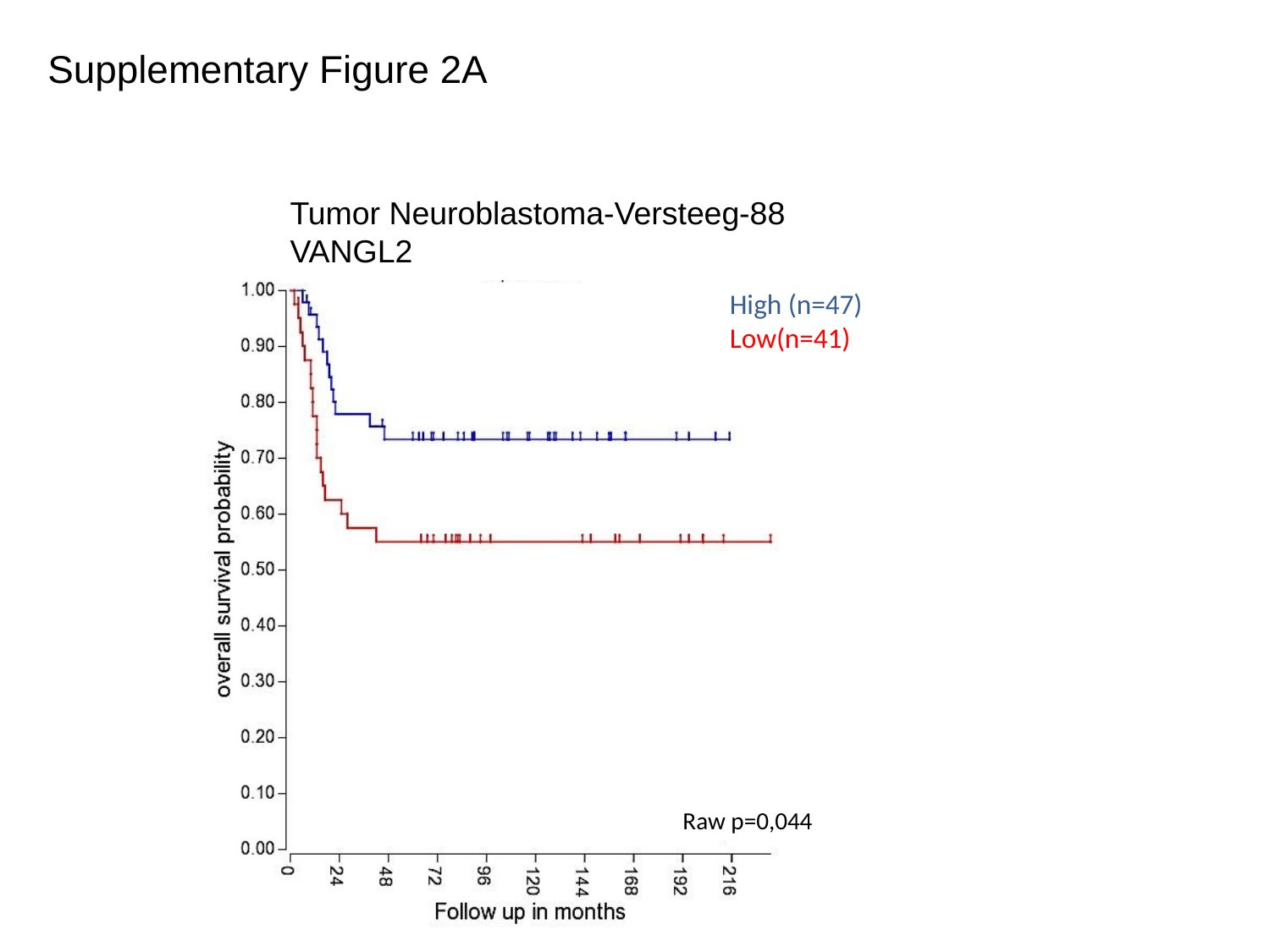

Supplementary Figure 2A
Tumor Neuroblastoma-Versteeg-88
VANGL2
High (n=47)
Low(n=41)
Raw p=0,044

## Slide 4
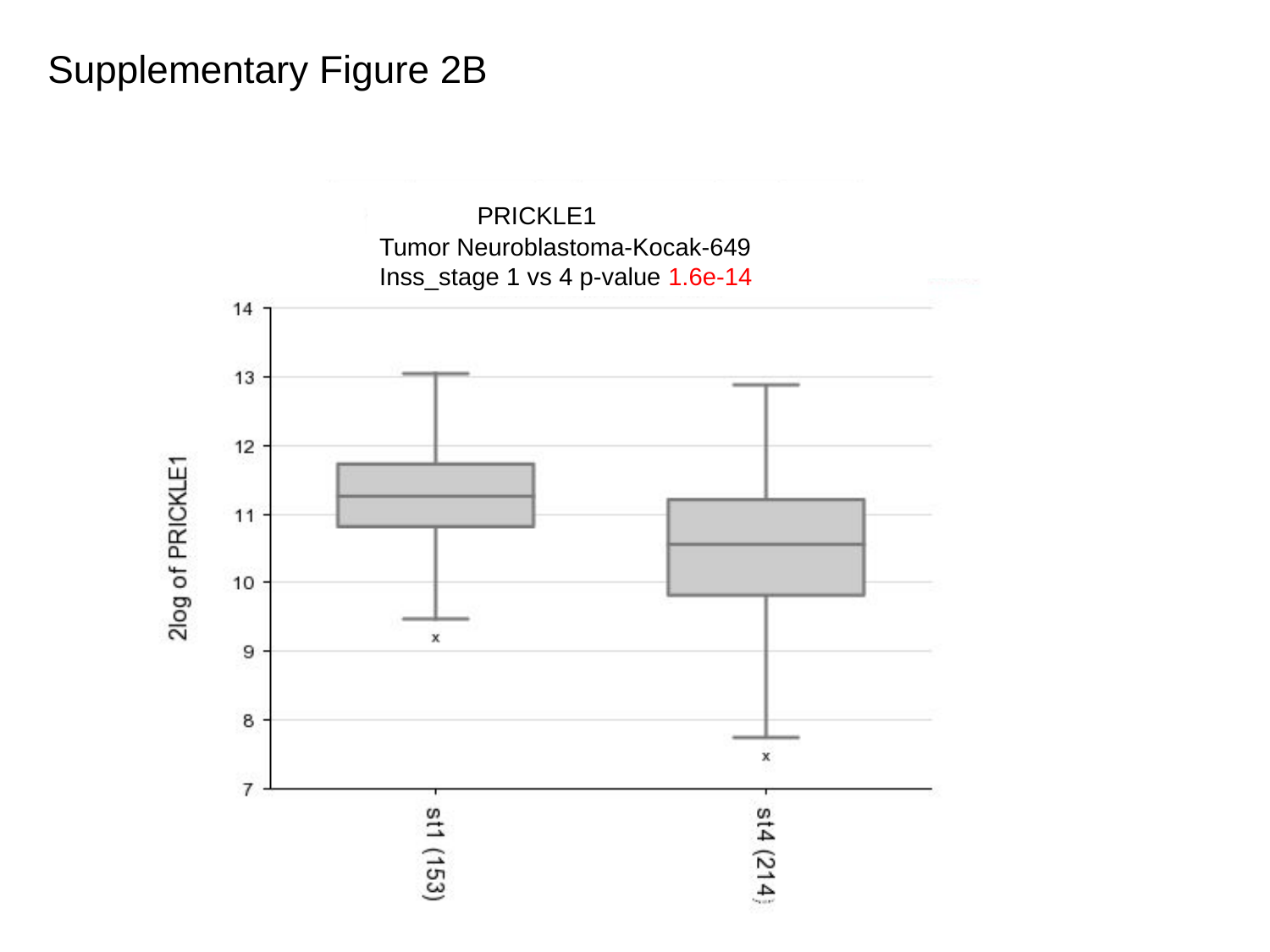

Supplementary Figure 2B
 PRICKLE1
Tumor Neuroblastoma-Kocak-649
Inss_stage 1 vs 4 p-value 1.6e-14
